# Stigma and social symptoms in Parkinson’s disease: A scoping review

**DOI:** 10.1016/j.prdoa.2026.100446

**Published:** 2026-05-06

**Authors:** Francesca Gaiera, Emma O’Shea, Gerard W. O’Keeffe, Mary Doherty, Suzanne Timmons

**Affiliations:** a Centre for Gerontology and Rehabilitation, School of Medicine, University College Cork, Ireland; b Department of Anatomy and Neuroscience, School of Medicine, University College Cork, Ireland; c Parkinson’s Disease Research Cluster, College of Medicine & Health, University College Cork, Ireland

**Keywords:** Parkinson’s disease, Enacted stigma, Felt stigma, Quality of life, Social symptoms, Isolation

## Abstract

**Background:**

The incidence of Parkinson’s disease (PD) is increasing more rapidly than any other neurodegenerative disorder. Motor and non-motor symptoms, and deficits in cognition and communication, negatively impact people with PD’s social functioning and can result in enacted and felt stigma. The aim of this scoping review is to explore the determinants and effects of stigma in PD.

**Methods:**

This scoping review followed the Joanna Briggs Institute methodology for scoping reviews. Quantitative and qualitative studies from January 2000 to April 2026 focusing on stigma in PD were considered for inclusion. Databases included Medline via OVID, PsychInfo and Scopus.

**Results:**

From 334 screened articles, 25 papers were finally included. Most papers found depression to be a determinant of enacted and felt stigma. Some papers found motor symptoms, younger age, difficulty with activities of daily living, and severe PD to be predictive of stigma. Facial masking, abnormal bodily movement, and speech impairment were linked to felt stigma and to stigmatising attitudes from the general public and clinical practitioners alike.

**Discussion:**

Stigmatising views have damaging consequences for people with PD. Negative outcomes of stigma include greater difficulties in the social and emotional spheres, and social avoidance and isolation. More awareness and education for the public, but also healthcare providers, are needed to address negative attitudes and bias towards people with PD. There needs to be more research into interventions to reduce felt stigma, such as psychological training or peer support.

## 1 Introduction

Neurodegenerative disorders are the leading cause of disability globally [1]. Parkinson’s disease (PD) is the second most common neurodegenerative disease after Alzheimer’s disease, affecting 2 to 3% of the population over the age of 65 [2]. The prevalence of PD is reportedly increasing more rapidly than any other neurological disorder [[3], [4]]. From 1990, the prevalence of people with PD increased 2.4 times, reaching 6.1 million in 2016 [3].

PD is characterised by motor symptoms (MS), such as bradykinesia, tremor, rigidity, and balance and coordination impairment [5], which can be obvious physical manifestations of PD, marking a patient out as different [6]. Beyond their neurological significance, these outwardly visible features function as social symptoms. A common motor feature of PD is hypomimia, or facial masking, due to facial muscle bradykinesia. This causes a frozen and unbroken stare and can create the image of an asocial, apathetic, and/or cold person [6]. The prevalence of facial masking ranges from 39% to 65% [7]. People with Parkinson’s (PwP) also express fewer and more ‘forced’ smiles than their healthy counterpart [8], a feature known to induce empathic and warm responses from others [9]. Simultaneously, impaired cognitive flexibility can compromise PwP’s ability to imitate or modulate facial expressions depending on the situation [10].

PwP also experience deficits in social cognition, which impairs their ability to properly perceive and interpret social stimuli [11], such as recognising and deciphering facial expressions, body language, and speech prosody, and, thus, properly responding to social cues. Social perceptual processing occurs in brain regions implicated in the degeneration of PD, including the basal ganglia, amygdala, and prefrontal cortex, which are involved in emotion recognition and social cognition [12]. Deficits in recognising facial expressions, particularly negative emotions, such as fear, anger, and disgust, have been reported in PD populations (e.g., [[13], [14], [15]], and are linked to facial masking. Indeed, imitating an expression can signal sensorimotor cues that provide feedback to the brain to support emotion recognition [16].

Some PwP can also experience disrupted speech patterns, such as dysarthria and dysphonia [6]. The person’s speech is grammatically and syntactically accurate, but accompanied by an irregular rhythm, a coarse voice, unexpected pauses, and the loss of prosody [17]. Together, these communicative and expressive changes represent social symptoms that may be misinterpreted by others as reflecting personality traits, such as disinterest, apathy, introversion, or reduced emotional responsiveness, rather than underlying neurological impairment [18].

Features of PD that affect social interactions have been reported to lead to social isolation for PwP [19]. Among the determinants of social isolation are facial masking [20] and speech impairment[21], which cause feelings of frustration and embarrassment and prevent PwP from being involved in social opportunities, leading to social disconnectedness. This isolation is not only detrimental to mental and physical health [6] but is also recognised as a significant risk factor for cognitive decline and dementia in older adults (e.g., [22].

Another variable implicated in PwP’s social isolation is stigma. Stigma is a phenomenon bred from the sociocultural process whereby members of minority groups are segregated and labelled as abnormal and unwelcome in society [23]. Drawing on Goffman’s theory of stigma [24], stigma emerges when a particular attribute comes to dominate social perception, leading the individual to be viewed as diminished or devalued rather than as a complete person. In PD, socially visible symptoms, such as facial masking or atypical speech, can become socially and morally salient cues that invite judgment, misinterpretation, and social distancing [[6], [25]].

‘Enacted’ stigma refers to the PwP’s experience of discriminatory behaviours from others, such as social exclusion, staring, avoidance, or intrusive questioning [25]. Internalised or ‘felt’ stigma refers to the process whereby the PwP endorses and applies negative stereotypes about the condition to themselves, thereby internalising these beliefs [25]. From a labelling perspective, repeated social responses to these symptoms may reinforce a stigmatised identity, shaping both how PwP are treated and how they come to see themselves.

PD is inherently characterised by the presence of non-motor symptoms (NMS) such as depression, anxiety, and apathy, which can precede the formal diagnosis by several years [26]. These symptoms can significantly impact emotional processing, motivation, and interpersonal engagement, often reducing an individual’s capacity to initiate or sustain social interaction. In particular, apathy and depression may contribute to diminished affective expression and withdrawal from social contexts, compounding the communicative difficulties already associated with the condition. As a result, NMS play a critical role in shaping both the lived experience of PD and how individuals are perceived by others, with important implications for psychosocial functioning and quality of life (QoL). However, stigma and loneliness may influence the QoL and life satisfaction to a greater extent than either MS or NMS [[6], [27], [28]]. Stigma, in particular, arises when visible and communicative features of the condition are socially misinterpreted, leading to negative judgments and altered interpersonal responses. These experiences can contribute to heightened self-consciousness, reduced self-esteem, and increased vulnerability to depression and anxiety. In turn, individuals may withdraw from social interaction to avoid perceived scrutiny or misunderstanding, reinforcing feelings of isolation and further exacerbating psychological distress. This suggests the presence of a cyclical process, whereby the manifestations of the condition contribute to stigma, which in turn amplifies emotional and social difficulties.

### 1.1 Aim

We hypothesised that stigma in PD can engender a loop of negative consequences, such as increased depression and anxiety, social avoidance, and isolation. The scoping review focused on the determinants and the effects of stigma in PD, specifically the role of facial masking, abnormal bodily movements (ABMs), and speech difficulties as risk factors for stigma.

### 1.2 Purpose

A greater understanding of the role of stigma in PD will lead to appropriately designed and implemented social and health policies, ultimately educating practitioners and the general public, with a view to dissolving the stigma associated with PD.

### 1.3 Methodology

**Fig. 1:**
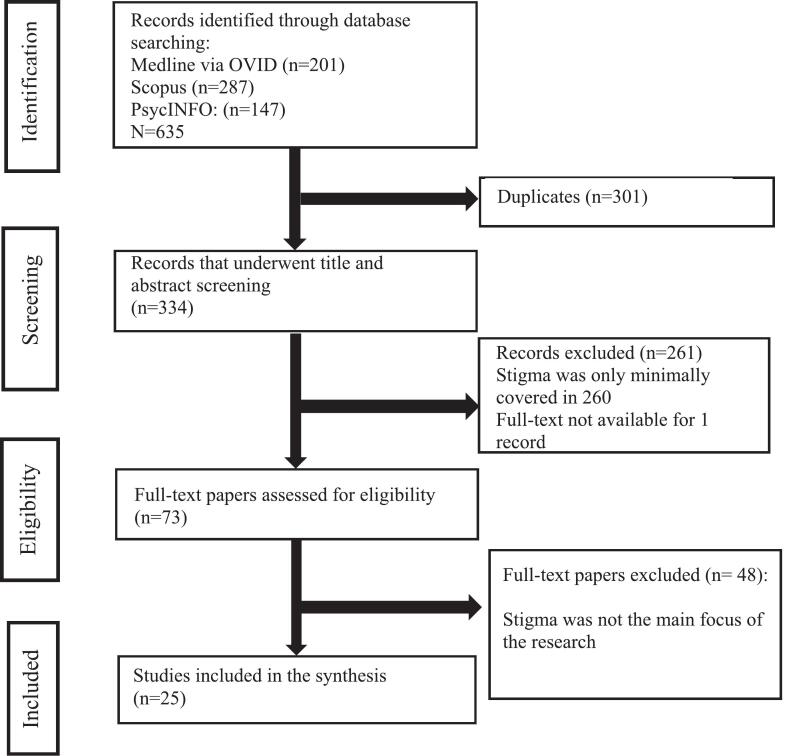
PRISMA flowchart.

This scoping review followed the Joanna Briggs Institute (JBI) methodology for scoping reviews and is reported in accordance with the PRISMA-ScR (Preferred Reporting Items for Systematic reviews and Meta-Analyses extension for Scoping Reviews) checklist to ensure transparent and comprehensive reporting [29]. The study selection process is summarised in a flow diagram (Fig. 1), and data extraction is presented in Table 2. The initial inclusion criteria were wide (Table 1). The review considered studies that explored the role of stigma in PD and socially-relevant symptoms of PD (i.e., facial masking, ABM, speech impairment) as risk factors for stigma.

**Table 1:** Inclusion and Exclusion criteria.

Inclusion criteria	Exclusion criteria
Research participants were PwP or their family members or the general population or healthcare professionals Study gave information on prevalence or consequences of either i) stigma in PD or ii) socially relevant PD features	Studies that do not primarily explore the roles of social symptoms and/or stigma in PD
Study involved primary data collection, using any study design Published between January 2000 and April 2024 Full-text paper was in the English language	Scoping reviews and systematic reviews, dissertations, conference reports, books, protocols, opinion pieces or commentaries Published before the year 2000 Publications in any other languages than English Full-text publications not available after contacting authors

**Table 2:** Summary of studies included in the scoping review.

**Study**	**Sample**	**Methodology**	**Country**	**Aim**	**Findings**
[30]	54 PwP (males = 34; females = 20). M Age = 58.2; MDD = 14y (complex motor stage); Mdn DD = not specified	Survey (quantitative) MDS-UPDRS, PDQ-39	Brazil	To identify the variables associated with stigma in PD patients who are candidates for deep brain stimulation (DBS).	More difficulties with activities of daily living (MDS-UPDRS III) was associated with greater stigma (PDQ-39) in PwP who are candidates for DBS, p = 0.048.
[31].	142 PwP (males = 57; females = 85). M Age = 62.98; MDD = not specified; Mdn DD = not specified	Survey (quantitative) PDQ-39, Chronic Illness Anticipated Stigma Scale, Hoehn and Yahr Scale, HDRS	Turkey	To assess the level of stigma and the factors influencing stigma among PwP.	Stigma (PDQ-39; Chronic Illness Anticipated Stigma Scale) was more prevalent in the tremor-dominant subtype and related to younger age, shorter disease duration, greater disability (Hoehn and Yahr scale), p = 0.023, and depression (HDRS), p = 0.000.
[32]	130 PwP (males = 56; females = 74). M Age = 64.68; MDD = 5.2y; Mdn DD = not specified	Survey (quantitative) SSCI, Self-Compassion Scale, DASS	UK	To assess the relationships between self-compassion, felt and enacted stigma and psychological distress among PwP.	Felt stigma (SSCI) mediated the relationship between self-compassion (Self-Compassion Scale) and depression, anxiety and stress (Depression, Anxiety, and Stress Scale; DASS). Self-compassion did not moderate the relationship between enacted stigma (SSCI) and depression, *p* = 0.905; or between enacted stigma and anxiety, (*p* = 0.875). or between enacted stigma and stress, *p* = 0.325.
[33]	55 PwP (males = 32; females = 23), M Age = 66.5, MDD = not specified; Mdn DD = not specified 23 caregivers (males = 7; females = 16), M Age = not specified	Interviews (qualitative)	Kenya	To highlight the interplay of structural constraints and the negative consequences of stigma experienced by PwP.	Stigma was found to impact social relationships, resource availability (social, material and financial), access to health services and medication, stress and psychological responses and health outcomes.
[34]	21 PwP (males = 11; females = 10) M Age = 61.6; M DD = 8.66; Mdn DD = 8	Interviews (qualitative)	Ireland	To explore the social experiences of PwP in Ireland, including the role of stigma.	PwP commonly experienced stigma in social situations (e.g., feeling pitied or misjudged). While most remained socially active with strong support networks, some avoided unfamiliar settings due to visible symptoms. Many reported difficulty sharing emotional experiences and a need for additional emotional support. Findings highlight stigma’s impact on well-being and the need for education and support services.
[18]	58 healthy participants (males = 18; females = 40). M Age = 75.76	Subjects rated (5-point Likert scale) observations of PwP based on relationship interest* and perceived positive behaviour (Positive and Negative Social Exchange Assessment).	US	To investigate differences in enacted stigma due to facial masking between observed men and women with PD.	Masking affected ratings of women with PD (N = 6; 50:50 low/high masking) on relationship interest, *p* < 0.01, social support, *p* < 0.01, but not men with PD (N = 6; 50:50 low/high masking). Authors suggested that higher masking violates gender norms for expressivity in women but not in men. *(“How likely do you think it is that you could have a happy/fulfilling social life with this person?” and “Would you be interested in getting to know this person better?”)
[35]	59 healthy participants (males = 24; females = 85) M Age = 76.37	Ratings of perceived social positivity and supportiveness (PD Q39) from observations of masking and ABM (quantitative)	US	To investigate stigmatising attitudes towards facial masking and ABMs of PwP.	Observers’ impressions were more negative for perceived social positivity**, ***p* < 0.001, and supportiveness**, ***p* < 0.001, when the subjects had either higher masking or ABM (24 x 20-sec clips; 6 = low masking, *p* < 0.001, and low ABM, *p* < 0.001, 6 = low masking and high ABM; 6 = high masking and low ABM; 6 = high masking and high ABM).
[36]	14 PwP (males = 7; females = 7) M Age = 68.4; MDD = not specified; MDN DD = not specified	Interviews and observations at 2 PD support groups (qualitative)	US	To explore felt stigma.	Felt stigma was associated with a changing self, and a form of disability and mounting isolation. Facial masking was identified as one of the visible features leading to felt stigma.
[37]	276 PwP (males = 141; females = 135). M Age = 62.5; MDD = 9.9y; Mdn DD = not specified	Survey (quantitative) UPDRS-III, HDRS, SSCI	China	To investigate the extent of enacted and felt stigma and its predictive factors in PwP.	Younger age, *p* < 0.043, motor symptoms (UPDRS-III), *p* < 0.001, and depression (HDRS), *p* < 0.001, were significantly correlated with enacted and felt stigma (SSCI).
[38]	200 PwP (males = 124; females = 76) M Age = not specified; MDD = not specified; Mdn DD = not specified	Survey (quantitative) SSCI, MDS-UPDRS	China	To assess levels of stigma and identify demographic and clinical factors associated with stigma in PwP.	Stigma (SSCI) was significantly associated with disease severity, with MDS-UPDRS scores positively correlated with total stigma (p < 0.001), internal stigma (p < 0.01), and external stigma (p < 0.01). Higher stigma scores were observed in younger patients, males, those with lower education, greater dependency, non-married status, longer disease duration, and tremor-dominant subtype. Disease severity and demographic factors were identified as independent predictors of stigma.
[39]	196 PwP (males = 81; females = 113; n/a = 2) M Age = 64.8; MDD = 5.2y; Mdn DD = not specified	Survey (quantitative) SSCI, The Stigmatization Scale, Mental Health Consumers’ Experience of Stigma Scale, PDQ-39, BDI, BDI-II, Parkinson’s Anxiety Scale	US	To investigate the impact of health conditions on perceived stigma and QoL in PwP.	Perceived stigma (SSCI; The Stigmatization Scale; Mental Health Consumers’ Experience of Stigma Scale; PDQ-39) and poorer QoL (PDQ-39) were associated with thyroid disease, *p* < 0.05, depression (BDI-II), *p* < 0.001, anxiety (BDI; Parkinson’s Anxiety Scale), *p* < 0.001, and the total number of health conditions (self-report), *p* < 0.001.
[40]	30 healthy people (males = 15; females = 15) M Age = 24.2	Ratings (scale) from audio clips of speakers and discourse characteristics	Canada	To investigate how speech patterns in PD contribute to linguistic and social impressions from the perspective of listeners.	PD speakers (N = 15; with low disturbance of speech intelligibility as rated by a speech pathologist) were perceived as less interested, less involved, less happy and less friendly than healthy speakers (N = 13), p < 0.001.
[41]	224 PwP (males = 121; females = 103), median age = 60.4; MDD = not specified; Mdn DD = 1.2y	Survey (quantitative) PDQ-39	China	To investigate the development and evolution of self-stigma in patients with early stage PD; to explore the associated and predictive factors of self-stigma	The prevalence of self-stigma (PDQ-39) decreased from 58.0% at baseline (N = 130) to 49.2% after 3 years (N = 96), p = 0.071. Depression was the only predictive factor.
[42]	347 PwP (males = 165; females = 180; prefer not to say = 2) M Age = 64.8; MDD = 5.4y; Mdn DD = not specified	Survey (quantitative) Becks’ Depression Inventory, SSCI, Stigmatization Scale and Mental Health Consumers’ Experience of Stigma Scale, Parkinson’s Anxiety Scale, BAI, MDS-UPDRS	US	To investigate younger age, depression, and anxiety as predictors of stigma perception.	Younger age, *p* < 0.0001, and depression (Becks Depression Inventory), *p* < 0.0001, were predicative of higher felt stigma (SSCI, Stigmatization Scale, and Mental Health Consumers’ Experience of Stigma Scale) in men; depression predicted higher felt stigma in women, *p* < 0.0001. Anxiety (Parkinson’s Anxiety Scale; BAI) and motor function (MDS-UPDRS) were also predictors of stigma in the whole sample.
[19]	73 PwP (males = 44; female = 29). M Age = 65.7; MDD = 8.3y; Mdn DD = not specified	Survey (quantitative- longitudinal) PDQ-39, GDS, MDS-UPDRS	US	To investigate the role of felt and enacted stigma in health-related QoL.	Both felt and enacted stigma (SSCI) made a significant contribution to QoL (PDQ-39), *p* < 0.001. Those reporting higher stigma tended to have more severe PD, greater depression (GDS) and MS (MDS-UPDRS). Felt stigma was experienced more than enacted, by most participants.
[43]	90 PwP (males = 56; females = 34) M Age = 65.5; MDD = 7.3y; Mdn DD = not specified	Survey (quantitative) SSCI, PDQ-39	US	To assess the relationship between facial masking and QoL in PwP.	PwP with more severe facial masking (self-report), especially women, feel more stigmatised (SSCI), *p* < 0.05, and have lower QoL (PDQ-39), *p* < 0.001.
[44]	7 PwP (males = 3; females = 4) M Age = 63.71; MDD = 12; Mdn DD = not specified. 5 caregivers (males = 1; females = 4) M Age = 61.8	Interviews (qualitative)	US	To explore how people with Parkinson’s (PwP) and caregivers perceive and experience different types of stigma, and to examine relationships between stigma types.	Multiple stigma types (public, self-stigma, stigma by association, and structural stigma) were experienced by PwP and caregivers. Stigma was evident across interviews, literature, and social media, with interrelationships identified between stigma types. All forms of stigma negatively impacted individuals, highlighting the role of low public awareness.
[45]	200 healthy participants (males = 74; females = 126)	Survey of: knowledge; perceived commonness and personal likelihood of PD, PD seriousness/unpleasantness, control over symptoms; worry about contracting it; beliefs about protective measures, and stigmatising beliefs.	Australia	To investigate stigma towards PwP.	There is significant stigma perceived to be associated with PD, as well as significant misconceptions about the course and outcomes of the disease.
[46]	362 PwP (males = 205; females = 157) M Age = 67; MDD = 6.1y; Mdn DD = not specified	Survey (quantitative) GDS, PDQ-39, MDS-UPDRS	US	To investigate the factors that contribute to self-perceived stigma.	Younger age (men), *p* < 0.001, and depression (men and women; GDS), *p* < 0.001, were the primary predictors of self-perceived stigma in PD (PDQ-39). MS (UPDRS) did not predict the experience of self-stigma.
[47]	20 healthy participant (males = 10; females = 10) M Age = 67	Ratings, by healthy controls, of video clips (including voice audio) of PwP and HC No standardised questionnaire used, rating scales included emotional expressivity, friendliness / warmth, likeability, social engagement, emotional involvement	Canada	To investigate the perceptions of healthy participants of PwP facial expressiveness and voice	PwP (males = 8; females = 9; M Age 64.7; MDD = 9.9y) were judged more negatively on the social desirability dimension compared to healthy controls (males = 10; female = 10; M Age 62.8).
[48]	80 healthcare workers (HCW): Novice (30 females; M Age = 22); Expert (males = 5; females = 45; M Age = 33)	Ratings (5-point Likert scale) on observations of PwP (males = 5; female = 1; 49–79 years old) for perceived extraversion. MDS-UPDRS, NEO-Five Factor Inventory	US	To examine novice and expert practitioners’ impressions of the personality of PwP.	HCWs: PT = 11 novice, 26 expert; OT = 11 novice, 18 expert; SLT = 7 novice, 5 expert; Medicine = 1 novice, 1 expert. HCWs, especially novices, appeared to be overly sensitive to expressive masking (UPDRS) when forming impressions about patient extraversion (NEO-Five Factor Inventory; 5-min clip; total clips = 7/8), p < 0.05. Facial expressions were rated on a 5-point Likert scale by the HCWs.
[49]	284 HCWs (males = 146; females = 138) M Age clinicians = 31.9; M Age students = 22.4	Ratings of observations (quantitative) GDS	US	To evaluate the effect of masking, culture and gender on practitioners’ impressions of PwP’s psychological attributes.	HCWs (clinicians = 159; students = 125) judged patients (N = 24) with higher masking to be more depressed (GDS), p < 0.0001, and less sociable, less socially competent, and less cognitively competent (“I feel the patient is [trait]; “I think the patient would like [activity].)”, p < 0.0001, than patients with lower masking and normal expression.
[50]	229 PwP (males = 113; females = 116). M Age = 65; M age at diagnosis = 60y; MDD = not specified; Mdn DD = not specified	Survey (quantitative) SSCI, Parkinson's UK Scale of Perceived Control; General Self-Efficacy Scale, Positive and Negative Affect Schedule, PDQ-39, DASS-21	UK	To investigate whether the perception of control mediates the relationship between stigma and well-being in PwP.	Stigma (SSCI) was negatively correlated with perceived control (Parkinson's UK Scale of Perceived Control; General Self-Efficacy Scale), p < 0.001, positive affect (Positive and Negative Affect Schedule), p < 0.001, and QoL (PDQ-8), p < 0.001., and positively correlated with anxiety (DASS-21), p < 0.001. Perceived control significantly mediated the relationship between stigma and QoL, depression and positive affect, p < 0.001.
[51]	9 PwP (male = 8; female = 1); MDD = not specified; Mdn DD = not specified. 9 spouses (opposite gender). Age categories (all): 30–49 (N = 2); 50–59 (N = 5); 60–69 (N = 10); 70–79 (N = 1)	Interviews (qualitative)	New Zealand	To provide an overview of patient and spousal experiences of living with a facial masking impairment in PD.	Key themes included the misidentification of masking as negative affect by the spouses, poor recognition of facial masking, and unmet health resource needs.
[52]	245 PwP (males = 134; females = 111) M Age = 67.12; MDD = not specified; Mdn DD = not specified	Survey (quantitative) Social Impact Scale, Health Literacy Scale, Family APGAR Index Questionnaire, Hospital Anxiety and Depression Scale,	China	To examine the mediating roles of health literacy and family function in the relationship between stigma and negative emotions in PwP.	Stigma (Social Impact Scale) was significantly associated with increased negative emotions (p < 0.01), and negatively associated with health literacy (p < 0.01) and family function (p < 0.01). Health literacy and family function both partially mediated the relationship between stigma and negative emotions.

M: Mean; MDD: Mean Disease Duration; PwP: People with Parkinson’s; HC: Healthy Controls; MDS-UPDRS: Movement Disorder Society – Unified Parkinson’s Disease Rating Scale; PDQ39: Parkinson’s Disease Questionnaire 39; HDRS: Hamilton Depression Rating Scale; SSCI: Stigma Scale for Chronic Illness; ABM: Abnormal Bodily Movement; BDI: Beck Depression Inventory; BAI: Beck Anxiety Inventory; MS: Motor symptoms; QoL: Quality of Life; GDS: Geriatric Depression Scale; HCWs: Healthcare Workers;

#### 1.3.1 Population

The population of interest was PwP, but the population in eligible papers could include PwP, and/or their families and carers, and/or the general public, and/or Healthcare Practitioners involved in the care or management of PD.

#### 1.3.2 Concept

All studies with a focus on any aspect of stigma in PD or the impact of socially relevant PD symptoms were eligible. We included felt (internal) and enacted (external) stigma. Socially relevant symptoms are facial masking, ABM, and speech impairment.

#### 1.3.3 Context

The scoping review aimed to explore the breadth and scope of the current literature published on stigma and socially relevant symptoms in PD. The studies could be in any country or setting, and could be qualitative, quantitative, mixed-methods, or observational studies.

Inclusion and exclusion criteria (Table 1).

### 1.4 Search terms

The keyword Parkinson* was used for all literature research. Additional keywords guiding the search were “*stigma OR stigmatisation OR stigmatization AND quality of life, social symptoms”.*

The search strings were “Parkinson*’ AND “stigma” AND ‘social’.

### 1.5 Databases searched

The literature review was performed on Medline via OVID, PsychInfo and Scopus databases between January 2024 and April 2026, with the last research dated the 5th of April 2026. Articles were limited to peer-reviewed journals in the English language.. If a publication was considered relevant from abstract screening, but access to the full-text was not available, authors were contacted to request the full-text article. Review articles, dissertations, conference papers, books, protocols, opinion pieces, commentaries, and other forms of secondary research were excluded to ensure that only primary studies were included in the synthesis, in line with guidance for scoping reviews aiming to map original evidence. While existing reviews can provide valuable summaries of the literature, their exclusion allowed for a more direct and transparent mapping of how stigma in PD has been investigated across individual studies.

### 1.6 Screening processes

Potential papers were identified after the exclusion of duplicates. Two researchers (FG and MF) independently screened these by reviewing titles and abstracts. Those papers whose title or abstract did not focus on stigma were *a priori* rejected. The two researchers met to resolve disagreements, with four studies requiring reconsideration; where consensus could not be reached, a conservative approach was adopted, erring on the side of inclusion. Potential articles remaining after this initial screen were read, again independently, in full, and any discrepancies in opinion about inclusion were discussed. Three studies required review by a third, senior researcher (EOS), who made the final decision.

### 1.7 Data extraction processes

Two researchers (FG and MF) performed an iterative procedure of re-reading, taking notes, and discussing each included paper. A data extraction table was built to collect extracted data under relevant headings.

Methodological quality of included studies was assessed using the JBI critical appraisal checklists appropriate to each study design. Appraisal was conducted to identify potential sources of bias, including sampling methods, measurement approaches, and control of confounding variables. The results of the appraisal informed the interpretation of findings but were not used to exclude studies, in line with scoping review guidance.

### 1.8 Analysis

Results were collated and synthesised and are presented narratively. The findings are also graphically summarised in Table 2.

## 2 Results

Database searching identified 635 papers. After removing duplicates, 334 records remained. Following title and abstract screening, 73 articles underwent full-text assessment, of which one was excluded due to lack of access. 25 studies published between April 2004 and April 2026 met the eligibility criteria and were included in the scoping review.

Most studies were conducted in the United States (n = 11), followed by China (n = 4), the UK (n = 2), and Canada (n = 2), and Australia, Brazil, Kenya, Turkey, Ireland, and New Zealand (n = 1 each). Samples were drawn from both PwP (males = 1219; females = 1151) and the general population (males = 424; females = 764; unknown = 34), resulting in a pooled sample of 3,594 participants.Nineteen studies focused primarily on stigma in PwP’s lives, while six examined PD-related features and symptoms associated with stigma. Twenty studies employed quantitative methods, including surveys (n = 14) and observational or audio-based ratings (n = 7), with sample sizes ranging from 14 to 377 participants. Five qualitative studies [[33], [34], [36], [44], [51]] used interviews and observations.

### 2.1 Overview of stigma in PD

Moore and Knowles [45] reported that 50% of participants of the general population perceived PD as a stigmatising illness, and those who endorsed this view held more negative attitudes towards the condition. Concerns focused on becoming a burden and on social judgement. However, no differences were found between stigmatisers and non-stigmatisers regarding perceived illness severity, associations with mental deterioration, or demographic factors.

In some regions, limited knowledge about PD contributed to stigma. Mshana et al. (2011) documented widespread misconceptions in Tanzania, where PD was sometimes associated with witchcraft or normal ageing, leading to discrimination and barriers to care. These beliefs were linked to fear, misunderstanding, and social exclusion.

Studies involving non-PD populations (Hemmesh, 2014; Hemmesh et al., 2009; [[40], [48], [49]]consistently found that PwP were perceived as less likeable than healthy counterparts by both the general public and healthcare professionals, reinforcing the social dimension of stigma.

### 2.2 Correlates of enacted or felt stigma

#### 2.2.1 *Depression and depressive symptom*s

Several studies examined psychological correlates of stigma. Salazar et al. [46], using survey-based measures including the PD Questionnaire-39 (PDQ-39) and the Unified PD Rating Scale (UPDRS), found that early-onset PD was associated with stronger feelings of stigma, particularly due to perceived disruption of family and occupational roles. Logan et al. [42] similarly reported that younger age predicted felt stigma in men, assessed through validated stigma scales, such as the Stigma Scale for Chronic Illness (SSCI), and related measures of psychological distress. Other studies, employing comparable survey-based approaches and instruments including the SSCI, Stigmatisation Scale, and PDQ-39, also identified higher levels of stigma among younger PwP [[31], [37], [39]]. Longitudinal evidence indicated that felt stigma was more prevalent in early disease stages. Lin et al. [41] reported a decline in felt stigma from 58% to 49% over three years, while Demiryurek and Demiryurek’s [31] cross-sectional study observed higher stigma in early-stage PwP.

Depression emerged as a consistent predictor of stigma. Salazar et al. [46] found significant associations between depression and felt stigma, particularly among men [r = 0.14]. Eccles et al. [32], using survey-based quantitative measures including the SSCI, the Self-Compassion Scale, and the Depression Anxiety Stress Scales (DASS), reported strong correlations between i) depression and felt [Spearman’s ρ = 0.68] and enacted stigma [Spearman’s ρ = 0.41]; ii) anxiety and felt [Spearman’s ρ = 0.58] and enacted stigma [Spearman’s ρ = 0.47]; and iii) stress and felt [Spearman’s ρ = 0.63] and enacted stigma [Spearman’s ρ = 0.47]. Islam et al. [39] identified the contribution of depression [r = 0.38], anxiety [r = 0.31 – 0.35], and morbidity (e.g., thyroid disease) [r = 0.25] to feelings of stigma, anxiety, and comorbid conditions as contributors to stigma. Lin et al. [41] and Logan et al. [42] similarly reported depression as a key determinant across genders.

Ma et al. [19] found that higher stigma was associated with increased depressive symptoms. Similarly, Demiryurek and Demiryurek [31], using measures including the PDQ-39 and Chronic Illness Anticipated Stigma Scale alongside the Hamilton Depression Rating Scale (HDRS), reported a significant association between stigma and depression (p = 0.000), with higher stigma also linked to greater disability and younger age. Zhu et al. [52] further demonstrated that stigma (Social Impact Scale) was significantly associated with increased negative emotions (p < 0.01), and negatively associated with health literacy (p < 0.01) and family function (p < 0.01). Health literacy and family function partially mediated the relationship between stigma and negative emotions, indicating indirect pathways through which stigma impacts psychological well-being.

In contrast, Da Silva et al. [30], in a sample of candidates for deep brain stimulation (DBS), did not observe a significant relationship between stigma and depression. In this study, stigma was assessed using the PDQ-39, while clinical severity was measured using the UPDRS; findings indicated that greater motor difficulties were associated with increased stigma (p = 0.048), but levels of depressive symptoms were comparatively low. This may reflect the specific clinical profile of DBS candidates, who are often carefully selected and medically optimised, potentially limiting variability in mood-related measures.

Overall, the literature largely supports a robust association between depressive symptoms and stigma, with discrepancies likely attributable to differences in sample characteristics and measurement approaches.

### 2.3 Quality of life

Given that both stigma and depression are known to adversely affect emotional well-being and social functioning, QoL provides an important indicator of the cumulative impact of these factors in PwP. Ma et al. [19], using the PDQ-39 to assess QoL, alongside the Geriatric Depression Scale (GDS) and the MDS-UPDRS, found that stigma was strongly associated with poorer QoL (r = 0.68), with felt stigma exerting a greater influence than enacted stigma. Verity et al. [50] similarly reported associations between stigma, reduced positive affect, and diminished QoL [r = 0.26].

Across studies, stigma was consistently linked to impaired emotional and social functioning[[19], [33], [36]]. Felt stigma was identified as the primary contributor to reduced QoL and increased social and emotional difficulties, including higher depression, anxiety, and reduced positive affect. These associations were measured using validated instruments, such as the SSCI, PDQ-39/PDQ-8 for QoL, the GDS, the DASS-21, and the Positive and Negative Affect Schedule (PANAS; [[19], [50]].

### 2.4 PD symptoms

Logan et al. [42] reported associations between felt stigma and both MS severity, measured using the MDS-UPDRS, and NMS, including depression (Beck Depression Inventory) and anxiety (Parkinson’s Anxiety Scale; Beck Anxiety Inventory). Similarly, Hou et al. [37] found that MS severity (UPDRS-III) and depression (Hamilton Depression Rating Scale) were significantly associated with both enacted and felt stigma (SSCI).In contrast, Da Silva et al. [30] found no relationship between stigma and MS. Demiryurek and Demiryurek [31] observed higher stigma among individuals with tremor-dominant PD, whereas Salazar et al. [46] did not identify MS as predictors of stigma. Huang et al. [38] reported that stigma (SSCI) was significantly associated with disease severity, with MDS-UPDRS scores positively correlated with total stigma (p < 0.001), internal stigma (p < 0.01), and external stigma (p < 0.01). Higher stigma scores were observed among younger individuals, males, those with lower education, greater dependency, non-married status, longer disease duration, and tremor-dominant subtype. Disease severity and demographic factors were identified as independent predictors of stigma.

These findings indicate variability in the relationship between stigma and clinical characteristics, including both symptom severity and disease subtype.

### 2.5 Other correlates

Additional predictors of felt stigma included difficulties with activities of daily living[[19], [39]], greater disease severity[37], lower educational attainment and comorbid health conditions[39]. Qualitative evidence indicated that stigma negatively affected social relationships and access to healthcare, contributing to stress, isolation, and poorer health outcomes[33]. Gaiera et al. [34] similarly highlighted the social impact of stigma, with PwP reporting experiences of being pitied, misjudged, or closely observed in social situations. While most remained socially engaged, some avoided unfamiliar contexts due to concerns about visible symptoms (e.g., eating difficulties, quiet voice, fear of falling). Although strong support networks were common, many participants reported difficulty expressing emotional experiences and a need for additional emotional support, including peer or professional interventions.

These findings are further supported by qualitative evidence from Mastel-Smith et al. [44], where individuals with PD reported experiences of social avoidance, negative public reactions, and even derogatory behaviours, such as receiving hostile comments or being judged when using disability parking. Participants also described how visible symptoms led others to assume cognitive impairment. Such experiences contributed to fear of stigma, reduced social interaction, and, in some cases, withdrawal from public life.

Most studies reported that felt stigma was more prevalent than enacted stigma[[19], [32], [36], [41], [42], [50]], and no study found enacted stigma to exceed felt stigma.

### 2.6 The effects of facial masking on stigma

Several studies suggested that impaired facial expressiveness and deficits in emotion recognition contributed to perceptions of PwP as cold, detached, or cognitively impaired (Hemmesh, 2014; [[47], [51]]. Facial masking was the most frequently examined symptom in stigma research, assessed using diverse methodologies, including observer-based ratings of perceived social traits (e.g., warmth, extraversion, and social engagement) from video or live observations (Hemmesh et al., 2009; [[47], [48]], self-report and clinical instruments, such as the PDQ-39, GDS, and MDS-UPDRS[43]; Hemmesh, 2014), and qualitative interviews and naturalistic observations in support group settings[[36], [51]].Facial masking was identified as a stronger predictor of low QoL than depression[43]. Authors proposed that reduced expressiveness led observers to infer low warmth, empathy, and social competence[36]; Hemmesh, 2014). Qualitative findings indicated that PwP perceived reduced expressiveness as contributing to social withdrawal and relationship difficulties [51]. Hemmesh (2014) reported that diminished facial expressiveness was associated with lower ratings of social positivity and competence. Absence of smiling was linked to negative personality attributions or assumptions of depression. Hermanns [36] found that PwP attempted to conceal symptoms or avoid social interactions due to perceived negative judgements, reflecting efforts to conform to societal norms and maintain social acceptance. Hemmesh et al. (2009) demonstrated that facial masking influenced observers’ willingness to form social relationships more strongly than perceptions of competence, particularly for women, who were judged more negatively than men. Schwartz and Pell [47] similarly reported more negative evaluations of PwP compared to healthy controls. Gender differences were observed, with women exhibiting facial masking being judged more negatively than men (Hemmesh et al., 2009; [43]. Consistent with these findings, women with PD reported greater relational difficulties and stigma[43].

Healthcare professionals were also influenced by facial masking. Tickle-Degnen et al. [49] found that clinicians and medical students perceived PwP with greater masking as more depressed, less sociable, and less cognitively capable. Cultural differences were observed between U.S. and Taiwanese practitioners, suggesting sociocultural influences on clinical impressions. American practitioners showed greater negative bias when judging sociability, whereas Taiwanese practitioners showed greater bias in evaluations of cognitive competence and social supportiveness; effects were also stronger for women. Wootton et al. [51] reported that facial masking disrupted emotional connectedness within romantic relationships. Partners often misinterpreted reduced expressiveness as emotional disengagement. PwP attempted to compensate through deliberate expressions, gestures, and touch, although these efforts were frequently experienced as effortful.

### 2.7 Abnormal body movement and speech impairment

Hemmesh et al. (2009) found that increased ABM, including tremor and uncoordinated movements, were associated with more negative social evaluations and reduced confidence in PwP’s functional abilities. These symptoms were also perceived as barriers to performing socially expected roles, particularly among women.

Speech impairment was examined by Jaywant and Pell [40] using rating scales based on audio recordings, assessing speaker and discourse characteristics. PwP were perceived as less happy, friendly, and emotionally engaged than healthy speakers, despite their speech being rated as more comprehensible and coherent. These findings suggest that stigma related to speech is driven by prosodic and expressive features rather than linguistic competence. Despite motor and vocal impairments, PwP were able to produce meaningful and structured conversational contributions that listeners recognised. Notably, only one study directly examined the role of speech impairment in stigma.

Based on JBI critical appraisal, study quality was variable. The evidence base was dominated by quantitative survey designs (approximately 75–80% of included studies), with fewer qualitative and observational approaches. Most studies were cross-sectional and relied on self-reported measures of stigma, quality of life, and psychological symptoms (e.g., PDQ-39, SSCI), increasing the risk of response and recall bias (e.g., [39]. Only a small number of studies employed qualitative methods or observational ratings. In addition, several studies did not clearly report sampling strategies or adequately control for potential confounding variables. The predominance of cross-sectional designs limits causal inference, and variation in measurement tools contributed to heterogeneity across findings.

## 3 Discussion

This scoping review examined the literature on stigma and socially relevant symptoms in PD, highlighting both felt (internalised) and enacted (external) stigma. Although the literature addresses these forms of stigma, there is limited exploration of how intrinsic (e.g., self-efficacy) and extrinsic mechanisms (e.g., social connectedness) shape the perception and experience of stigma. Societal beliefs create environments in which derogatory labels are internalised, contributing to felt stigma. Stigma, alongside core PD symptomatology, imposes a substantial burden on PwP, adversely affecting social and emotional well-being, and reducing QoL through social avoidance, isolation, and shame around symptoms such as facial masking, ABM, or dysarthria.

The included studies demonstrated a high prevalence of both enacted and felt stigma. Overall, felt stigma was more common and had a greater impact on social and emotional well-being than enacted stigma[[19], [41], [46]]. This finding is significant, suggesting that the anticipation of social judgment and rejection can influence behaviour and self-esteem, even in the absence of direct discriminatory experiences. PwP may withdraw from social situations or internalise negative beliefs about themselves, amplifying psychological burden.

Depression consistently emerged as a predictor of felt stigma. All but one study[30], which excluded participants with significant depressive symptoms, found positive associations between depression and stigma[[19], [46]]. Anxiety and psychological stress were also identified as predictors of felt stigma[[32], [46]]. Moreover, felt stigma was reported to be higher in early disease stages, a finding that may be expected given that individuals are still adjusting to diagnosis and may experience greater disruption to identity, social roles, and expectations, particularly in early-onset PD. Evidence also suggests that stigma may be elevated in specific clinical presentations, such as tremor-dominant subtypes, where visible symptoms are more salient. These findings highlight the importance of early psychosocial interventions, such as psychoeducation, cognitive–behavioural approaches, and peer support, to mitigate the impact of stigma on QoL, social engagement, and emotional health.

The evidence suggested facial masking is implicated in the experience of stigma. However, the distribution of evidence across social symptoms was uneven. Influences of facial masking on others (six studies) have been explored more than ABM (two studies) or speech disturbances (one study). As such, conclusions regarding the role of ABMs and speech impairment must be interpreted with caution, as the current literature base is comparatively limited and less direct. Importantly, this imbalance in the literature reflects a broader research focus on facial expressivity in PD, rather than an absence of stigmatising effects associated with ABMs or speech impairment. Identifying such gaps is a key contribution of scoping reviews and highlights priority areas for future research. Facial masking conveys reduced emotional expressivity, which observers frequently interpret as lack of warmth, empathy, or social competence[49]. Both the general public and healthcare professionals exhibited stigmatising attitudes toward PwP with facial masking, while PwP reported social withdrawal, avoidance, and embarrassment, reflecting felt stigma. Women with facial masking were judged more negatively than men, likely due to gendered expectations of expressivity and emotional responsiveness[53]; Hemmesh et al., 2009; [43]. Cultural context may moderate the experience and impact of stigma, as suggested by Wang et al. [54]. In communal societies, strong family ties, extended social networks, and shared caregiving responsibilities can buffer against social isolation and reduce internalised stigma. In contrast, more individualistic cultures may provide less social support, potentially amplifying the negative effects of visible symptoms and social withdrawal. ABM was linked to perceptions of physical incapacity, with men often judged as unable to perform instrumental tasks, and women as unable to fulfill domestic or personal care roles (Hemmesh et al., 2009). However, the empirical evidence for ABM-related stigma remains limited, and much of the current understanding is inferred rather than directly tested. Similarly, research on speech impairment (hypophonia, dysarthria, slow speech) is sparse. Despite this, findings suggest that stigma arises not from linguistic deficits per se, but from altered prosodic and expressive features, which may lead to misinterpretations of emotional engagement. A notable gap in the literature is the role of social connectedness in mitigating stigma and its consequences. Social connectedness fosters a sense of belonging, reinforces social identity, and provides emotional support, buffering against stressors[[55], [56]]. Evidence from chronic disease populations, including cancer, coronary heart disease, and rheumatoid arthritis, indicates that social support reduces morbidity and psychological stress (Brummet et al., 2001; [[57], [58]]. Conversely, social isolation is associated with adverse health outcomes, including an increased risk of dementia, particularly among older adults[59]; Livingston, 2020). PwP are particularly vulnerable to isolation due to disease progression, mood disorders, apathy, and feelings of shame or embarrassment[55]. Promoting social engagement and supportive relationships, both within community settings and through clinical or therapeutic interventions, may enhance coping, reduce felt stigma, and encourage participation in the community.

## 4 Strengths and limitations

This review followed the Joanna Briggs Institute methodology for scoping reviews, covering literature from January 2000 to April 2026 and including studies of PwP of any type, severity, or setting, as well as studies including spouses, healthcare professionals, or the general public. Broad inclusion criteria enhanced the comprehensiveness and inclusivity of the review.

Methodological limitations were identified. Many studies relied on self-report measures, which may be subject to response bias and limit reliability[39]; Hemmesh et al., 2009). Short questionnaires restricted variability, potentially influencing reporting on depression, symptom severity, facial masking, and QoL[[41], [46]]. Sampling bias was another limitation, with most participants drawn from predominantly Western and White populations, and only a small number of studies conducted in non-Western contexts (e.g., Kenya and Turkey), thereby reducing cultural diversity and limiting the generalisability of findings across cultural settings. Future research should prioritise the inclusion of more culturally diverse samples and cross-cultural investigations to improve the applicability of findings to broader populations. One study was initially excluded due to lack of full-text access; repeated attempts to contact the corresponding author were unsuccessful, and its potential influence on the findings is acknowledged. Furthermore, several studies employed experimental designs using videos or structured tasks (Hemmesh et al., 2009; [49], which may not reflect real-world social interactions. Finally, a further limitation is the exclusion of review articles and theses, which may have reduced the opportunity to integrate higher-level syntheses of the evidence. Future work could build on the present findings by incorporating systematic reviews or *meta*-analyses to deepen understanding of stigma-related processes in PD.

## 5 Conclusion and implications

Stigma in PD is prevalent, associated with depression, disease severity, younger age (including early-onset PD), MS, and reduced QoL. Facial masking is a key determinant of stigma, affecting public perception, relationships, and healthcare provider attitudes. While pharmacological and surgical interventions address visible symptoms, awareness of invisible burdens, including stigma, remains limited among practitioners. Interventions to reduce stigma, support QoL, and promote social engagement are warranted. This may include public education, clinician training, peer support, and psychological interventions to foster self-acceptance and coping.

Future research should address gaps in understanding the stigma associated with ABM and speech impairment and explore the protective effects of social connectedness. Incorporating perspectives of PwP and their communities will strengthen insights into lived experiences and guide the development of interventions to reduce felt stigma and improve psychosocial outcomes.

## CRediT authorship contribution statement

**Francesca Gaiera:** . **Emma O’Shea:** Writing – review & editing, Validation, Supervision. **Gerard W. O’Keeffe:** . **Mary Doherty:** Visualization, Software, Data curation. **Suzanne Timmons:** Validation, Supervision, Funding acquisition.

## Funding

The following publications have received funding for their work by:

1. [30]: PRONEX Program (Programa de Núcleos de Excelência – NENASC Project) of the Fundação de Amparo à Pesquisa e Inovação do Estado de Santa Catarina (FAPESC), and the Conselho Nacional de Desenvolvimento Científico e Tecnológico (CNPq), Santa Catarina, Brazil;
2. [33]: Economic and Social Research Council (ESRC), Northern Ireland and North East Doctoral Training Partnership (NINE DTP);
3. [39]: the American Parkinson’s Disease Association, the Boston University Alzheimer’s Disease Center and the Swiss National Science Foundation;
4. [40]: the Canadian Institute of Health Research, Institute of Aging and by the Fonds de la Recherche en Santé du Québec;
5. [41]: the National Key Research and Development Program of China, the 1.3.5 project for disciplines of excellence (West China Hospital, Sichuan University), the Science Foundation of Chengdu Science and Technology Bureau and the funding of the National Science Fund of China.
6. [19]: the National Institute of Nursing Research and the United States National Institutes of Health;
7. [45]: Swinburne Alumni and Parkinson’s Victoria;
8. [46]: by the National Institute of Neurological Disorders and Stroke, the American Parkinson Disease Association, Building Interdisciplinary Research in Women's Health, the Davis Phinney Foundation and the Parkinson Disease Foundation;
9. [47]:  Parkinson Society Canada/CIHR Institute of Neurosciences, Mental Health and Addiction Partnership Award;
10. [48]: the Royal Center for Late-Life Enhancement and the Center for Neurorehabilitation, both of Sargent College of Health and Rehabilitation Sciences, Boston University;
11. [49]: the National Institute of Neurological Disorders & Stroke of the U.S. National Institutes of Health under the Fogarty International Center’s Stigma and Global Health Research Program;
12. [51]: the Bryant Trust Postgraduate Research Scholarship and a University of Waikato Doctoral Scholarship;
13. [34]: Cork Branch of the Parkinson’s Association of Ireland;
14. [44]: University of Texas at Tyler Institute for Integrated Health;
15. [[32], [36]]; Hemmesh, 2014; Hemmesh et al., 2009; [[31], [37], [38], [42], [43], [50], [52]] reported no funding was associated with their work.

The present scoping review was funded by the Parkinsons Ireland Cork branch and the Parkinson’s Disease Research Cluster at University College Cork.

Clinical impact statement.

Findings from this scoping review showed a high incidence of enacted and, particularly, felt (subjective) stigma in people with PD. Facial masking, abnormal bodily movement and speech impairment were the key PD features related to stigma. The experience of stigma was linked to motor and non-motor symptoms, depression, quality of life, age and illness severity. Symptoms that affected social interactions caused felt stigma and influenced relationships and attitudes from the public and healthcare providers. Thus, there needs to be more awareness raising and education for the public and practitioners to address negative attitudes and bias towards PwP.

## Declaration of competing interest

The authors declare the following financial interests/personal relationships which may be considered as potential competing interests: Mindful that our identities can influence our approach to science, the authors wish to provide the readers with information on our backgrounds. With respect to our positions towards Parkinson’s disease, three authors have been part of the PD Research Cluster of the University College Cork, which endeavors to engage in research to better understand PD. One author was also part of the Committee of the Parkinson’s Association of Ireland.

All data have been made publicly available at the [repository name] and can be accessed at [persistent URL or DOI].
